# Scalable Adaptive Graphics Environment: A Novel Way to View and Manipulate Whole-Slide Images

**DOI:** 10.1155/2014/517953

**Published:** 2014-12-29

**Authors:** Victor Mateevitsi, Bruce Levy

**Affiliations:** University of Illinois at Chicago, Chicago, IL 60612, USA

## Background

The Scalable Adaptive Graphics Environment (SAGE) was developed at the University of Illinois at Chicago's (UIC) Electronic Visualization Laboratory (EVL) to facilitate collaborative efforts that require the sharing of data-intensive information for analysis. SAGE is a cross-platform, community-driven, open-source visualization and collaboration tool that enables users to access, display, and share a variety of data-intensive information, in a variety of resolutions and format, from multiple sources, on tiled display walls of arbitrary size. SAGE walls have had the ability to display digital-cinema animations, high resolution images, high-definition video-conferences, presentation slides, documents, spreadsheets, and computer screens; however, there was no way to display and manipulate histologic whole-slide images (WSIs). Our desire was to create a tool to permit the importation, display, and manipulation of WSI in the SAGE environment.

## Methods

The Pathology Department and the EVL at UIC through a grant from the University's Center for Clinical and Translational Science set out to develop a prototype of a SAGE tool for the use of WSI in this collaborative environment and then to create a series of scenarios for its use involving patient care, medical education, and research. The goal was to create a tool that would allow the user to manipulate the WSI in ways similar to common WSI viewers.

## Results

We were able to develop a usable prototype of a SAGE tool for the use of WSI. Our initial prototype can import only the NDPI file format utilized by Hamamatsu, which we chose as our primary scanner is a Nanozoomer. Once the WSI is imported, the tool allows the manipulation of the WSI (panning and zooming) from a computer through SAGE Pointer. A very simple annotation tool has been created as a prototype. We can open multiple WSI images simultaneously on six by three screen tiled display (the equivalent of a 250-inch monitor). Demonstration scenarios have been created for its use in patient care, medical education, and research. The very high resolution of this tiled display allows for detailed examination of the WSIs.

## Conclusions

SAGE is an ideal environment to display WSI for patient care, education, and research. We have demonstrated its use in patient care, education, and research. While our tool is merely a prototype at this time and only basic functionality, we hope to expand the tool in the future. We will expand the WSI file formats that can be imported to make the tool vendor neutral. We will increase the versatility of the annotation tools. We will create a mechanism for manipulating the WSI directly on the screen through the touch interface. We will explore the collaborative advantages of this tool through sending them between SAGE walls.

